# Lecture on Extra-Uterine Pregnancy*Delivered before the Students of the Atlanta Medical College, October 13th, 1894.

**Published:** 1894-12

**Authors:** James A. Goggans

**Affiliations:** Alexander City, Ala., Senior Counselor of the Medical Association of the State of Alabama; Fellow of Southern Surgical and Gynæcological Society; Fellow of the British Gynæcological Society


### ORIGINAL LECTURE ON EXTRA-UTERINE PREGNANCY.*

BY JAMES A. GOGGANS, M. D., OF ALEXANDER CITY, ALA.,

Senior Counselor of the Medical Association of the State of Alabama; Fellow of Southern Surgical and Gynæcological Society; Fellow of the British Gynæcological Society.

*Gentlemen*—It is through the courtesy of your Professor of Surgery that I have the honor of speaking to you to-day, and I wish to assure you that it is a privilege that I appreciate in the highest degree possible. I shall proceed at once to speak to you of a disease of great importance, a disease which any practitioner of medicine is liable to encounter, and with which we must deal promptly. Therefore, much more valuable information may yet be acquired by studying the clinical history of each case. I refer to *extra-uterine* pregnancy. This specimen which I show you is a three-months fœtus and the ruptured tube in which it was imprisoned. It was removed from a woman 35 years of age, and she presented the following history:

She had had three children, the youngest 5 years of age. Since the birth of her last child, she had had two abortions. I saw her in consultation August 31st, and she had had three attacks of severe pain in the lower abdomen, followed by complete prostration and a rapid pulse, and presenting all the symptoms of internal hemorrhage. Her menses had been regular, but she failed to menstruate on July 23d, at which time the menses was expected to appear. On August 15th, she passed a membraneous mass which was accompanied by the discharge of some blood; there was not much pain.

On making a vaginal examination, it was found that some blood was still passing, and a small, irregular tumor could be felt low down in the pelvis to the left side of and behind the uterus. This tumor was fixed and very painful on pressure. Taking all of these symptoms together, only one pathological condition could scarcely be thought of, viz.: tubal pregnancy, tube already having ruptured.

An operation for its removal was proposed, but she declined to have it done unless the above symptoms should arise again. She grew somewhat better for a week or two, and was able

*Delivered before the Students of the Atlanta Medical College, October 13th, 1894.

to walk about her room, but was again attacked with the same symptoms, and was brought to me a distance of fifteen miles for operation. The section was made on September 28th. On opening the abdomen, blood welled up in the incision, and the neoplasm was found bound down by many adhesions. The fœtus and placenta were still imprisoned in the tube, and a rupture had taken place through which I could pass my three fingers. This mass was tied off near the uterus, and the hemorrhage from the bottom of the pelvis was controlled by the free use of hot water and iodoform gauze packing. I believe this to be a disease of far more frequent occurrence than it is generally supposed to be, and I wish to refer again briefly to the symptoms which will usually present themselves in cases of *extra-uterine pregnancy*. I shall not attempt to describe to you the symptoms of all the generally recognized varieties of ectopic gestation, and will content myself by telling you that I believe with Mr. Doran, and Mr. Tait, that the original seat of all fœtal cysts is the tube.

We must remember that the symptoms of ordinary uterine pregnancy are not always constant. Neither are they constant in extra-uterine pregnancy. Menstruation at first usually disappears and then returns, and may even become continuous, accompanied by the expulsion of a decidual membrane. Besides this, we may have the other ordinary symptoms of uterine pregnancy, including enlargement of the uterus. The diagnosis, however, is based mainly upon the presence of a soft, fluctuating tumor lying to one side of, or behind the uterus, which grows gradually, and is more or less fixed and painful on pressure. Finally, rupture of the sac, accompanied by extreme anæmia and severe pain, and in many cases death soon follows. One other point to which I wish to call your attention especially, is the fluctuation of which I have already spoken in the cysts of extra-uterine pregnancy. Fluctuation cannot exist as a symptom in these cysts in the cases where rupture has already taken place.

And in the case of the woman from whom I removed this specimen fluctuation was not present. The contents of the cyst had already escaped into the abdominal cavity, hence the tumor was solid, irregular, fixed and painful. The etiology of the disease is still very obscure and will certainly remain so until the physiology of impregnation is better understood. The prognosis is very grave, so much so, that we are justifiable in regarding extra-uterine pregnancy as a most deadly disease. As to treatment, when left to nature, the outcome is very uncertain. Three-fourths of all cases die, and most of them from rupture of the cyst. The blood may be discharged through the tube into the abdominal cavity, known as *tubal abortion*, or the tube may be ruptured so that the blood may be discharged directly into the abdominal cavity, or into the broad ligament. However this may be, death takes place from anæmia or peritonitis, but the cause of such peritonitis is unknown. From what I have said regarding prognosis, you will perceive that the *treatment* of all forms of *ectopic gestation* consists in removing the neoplasm by abdominal section as early as possible after the diagnosis is made. Much has been said about treating this disease by injecting morphine into the sac, and about destroying the life of the fœtus with electricity. I must say that I regard those measures as unsurgical and fraught with much danger. Only eight or ten cases of extra-uterine pregnancy have been carried to full term with satisfactory results to both the mother and child, so the chances for such a happy result are so slender that I advise such patients to have the whole mass removed just as soon as the diagnosis is made certain. Especially is this treatment indicated during the first three months, and if the rupture has already taken place. After the fourth month and up to the period of false labor, operation is not advisable. Operate in all cases after false labor and death of the child, when the amniotic fluid has been absorbed, indicating that the circulation in the placenta has ceased. Under any circumstances I would advise operation when the life of the mother is in danger.

---

SALINES operate in three or four hours. Croton oil in one or two hours. Jalap, gamboge and senna in three or four hours. Rhubarb and castor oil in from four to six hours. Aloes and mandrake in from ten to fourteen hours.

